# Salivary Diagnostic for Monitoring Strenuous Exercise—A Pilot Study in a Cohort of Male Ultramarathon Runners

**DOI:** 10.3390/ijerph192316110

**Published:** 2022-12-01

**Authors:** Josephin Borchers, Cordula Leonie Merle, Deborah Diana Schöneborn, Lea Ronja Lyko, Thomas Thouet, Bernd Wolfarth, Tanja Kottmann, Carmen Scheibenbogen, Jeannot Zimmer, Sven Diederich, Katrin Bauer, Ulrich Sack, Gerhard Schmalz, Dirk Ziebolz, Jan Wüstenfeld

**Affiliations:** 1 Department of Prosthetic Dentistry, UKR University Hospital Regensburg, 93053 Regensburg, Germany; 2 Department of Cariology, Endodontology and Periodontology, University of Leipzig, 04103 Leipzig, Germany; 3 Department of Sports Medicine, Charité University Medicine, 10115 Berlin, Germany; 4 Department of Sports Medicine, Institute for Applied Scientific Training, 04109 Leipzig, Germany; 5 Institute of Sports Science, Humboldt University, 10115 Berlin, Germany; 6 CRO Dr. med Kottmann GmbH & Co. KG, 59077 Hamm, Germany; 7 Institute for Medical Immunology, Charité University Medicine, 13353 Berlin, Germany; 8 SYNLAB Medizinisches Versorgungszentrum Berlin GmbH, 10828 Berlin, Germany; 9 Medicover Berlin-Mitte, 10117 Berlin, Germany; 10 Institute of Clinical Immunology, Medical Faculty, University of Leipzig, 04103 Leipzig, Germany

**Keywords:** endurance, saliva, immunoglobulin, inflammation, cytokines, biomarkers

## Abstract

Intense physical stress, such as that in ultramarathon running, affects the immune system. For monitoring in sports medicine, non-invasive methods, e.g., salivary analysis, are of interest. This pilot cohort study aimed to assess changes in salivary parameters in response to an ultramarathon. The results were compared to blood parameters. Male, healthy finishers (*n* = 9, mean age: 48 ± 8.8 years, mean height: 1.8 ± 0.1 m, mean weight: 72.5 ± 7.2 kg, mean BMI: 23.5 ± 1.9 kg/cm²) of a 160 km ultramarathon were included. Saliva and blood samples were collected at three time points: T1 (baseline), T2 (shortly after the ultramarathon) and T3 (after recovery). In saliva, cortisol, testosterone, IL-1β, IL-6, IL-8, IL-10, TNF-α, albumin, IgA, α-amylase, aMMP-8, and neopterin were assessed via ELISA. In blood, cortisol, testosterone, IL-1β, IL-6, IL-8, IL-10, TNF-α, blood cell counts, procalcitonin, CRP, osmolality, albumin, and α-amylase were analyzed. The statistical evaluation comprised longitudinal testing and cross-sectional testing between saliva and blood using ratios of T2 and T3 to baseline values. Various parameters in saliva and blood changed in response to the ultramarathon. Comparing blood and saliva, the longitudinal changes of testosterone (*p* = 0.02) and α-amylase (*p* = 0.03) differed significantly. Despite the limitations of the study, it underlines that saliva is an interesting option for comprehensive monitoring in sports medicine and necessitates further studies.

## 1. Introduction

Physical stress caused by intense exercise has various effects on the immune system [1,2,3,4]. Previous studies have monitored the effects of exercise on various blood parameters, including Interleukin (IL) IL-1β, IL-6, IL-8, IL-10 and tumor necrosis factor alpha (TNF-α) [5,6]. These cytokines are inflammatory mediators of the immune system. The macrophage-derived mediator neopterin increases during training, too [7]. Furthermore, hormone levels change: cortisol increases and testosterone decreases [8,9]. However, blood sampling is an invasive method and requires medical expertise. Moreover, it can cause pain and stress.

How to provide non-invasive monitoring of exercise is an ongoing question in sports medicine. One promising medium for this aim is saliva [10]. Saliva contains antibodies, growth factors, proteins, organic non-protein compounds, hormones, and specific salivary enzymes [10]. Fluctuations of the anabolic hormones cortisol and testosterone in saliva [3] and the correlation of these parameters measured in blood and saliva were reported [11,12]. Elevated levels of salivary IL-6 [13] and TNF-α [14] were observed. Studying parameters in saliva offers additional advantages. For salivary IgA, a decrease was observed in response to strenuous exercise and over a longer period of strenuous exercise, such as a football league season [15]. This could lead to reduced mucosal immunity and a higher incidence of infections [16,17,18]. Thus, its monitoring was discussed to prevent infections [16,17,18]. Salivary α-amylase increased in response to exercise [19,20] and could sensitively reflect psychophysiological stress [21]. Furthermore, salivary active matrix metalloproteinase-8 (aMMP-8) was examined because it is involved in extracellular breakdown and is used as a marker for periodontal degradation [22].

The present pilot study aimed to provide a comprehensive evaluation of changes in salivary parameters due to intense physical stress (ultramarathon), as well as relation of salivary parameters to simultaneous blood parameters. It was hypothesized that both blood and salivary parameters would differ significantly between baseline and after an ultramarathon or in the recovery phase. Additionally, it was hypothesized that there would be consistent changes in blood and saliva regarding the ratios of T2 (shortly after the ultramarathon) and T3 (after recovery) to baseline values.

## 2. Materials and Methods

### 2.1. Study Design

The present pilot study was part of a clinical cohort study. The study was authorized by the ethics committee of the Charité University Medicine Berlin (registration number: EA2/107/16). The investigation took place during the 160 km ultramarathon “Berliner Mauerweglauf” in 2016 in Berlin, Germany. All registered participants were contacted via e-mail. Interested runners were informed verbally and in writing about the study and all included runners provided their written informed consent. The STROBE guidelines for reporting of cohort studies were followed [23]. All samples were collected at three time points: T1 as baseline (4–13 days before the ultramarathon, between 8 a.m. and 4 p.m., at the Department of Sports Medicine, Charité University Medicine, Berlin), T2 shortly after the ultramarathon (13–14 August 2016, within 30 min after destination entry, at the destination) and T3 after recovery period (8–11 days post run, between 8 a.m. and 4 p.m., at the Department of Sports Medicine, Charité University Medicine, Berlin). T1 and T3 took place on an empty stomach and participants were advised to avoid intense sporting 48 h before T1 and T3.

### 2.2. Participants

Inclusion criteria were participation in the ultramarathon, written informed consent, German-speaking, male, participation in the three examinations, age over 18 years, no cardiovascular diseases that lead to limitations for sporting, no diabetes mellitus, and no permanent medication. Participants that did not finish the ultramarathon as well as participants with missing saliva samples at any time point were excluded from the analysis.

In total, 22 male athletes were recruited. One athlete withdrew from the ultramarathon, two were excluded due to medical exclusion criteria. Consequently, 19 were included in baseline examinations, but five did not finish the ultramarathon and five did not provide complete saliva samples. Finally, nine runners were included in the present analysis. Table 1 shows the main characteristics of all participants. The mean age of the participants was 48 years (±8.8). The mean body fat percentage was 16.65 % (± 4.93). The mean running time was 23:29 h, indicating a mean pace of 08:42 min/km, and, respectively, a speed of 6.9 km/h.

### 2.3. Data Collection

A physical examination was performed including measurement of body composition (height, weight, body mass index, body fat percentage by bioelectrical impedance analysis) before the marathon (T1). Total running time and pace were measured during the ultramarathon. Saliva and blood samples were obtained from all participants at all three time points (T1–T3). All samples were transported in frozen status.

Saliva sampling [24]: Non-stimulated saliva was collected by cotton salivettes (Sarstedt, Numbrecht, Germany) for 2 min without chewing. Salivettes were directly centrifuged by 1000× *g* for 2 min (20 °C). These salivary samples were stored immediately on dry ice and then deposited at −80 °C at the University medical center Berlin Charité. 

Blood sampling [25]: Blood was collected from a peripheral vein of the sitting or lying participant using the vacutainer system and was processed on-site. For cytokine measurement, a serum monovette (9 mL, Sarstedt, Numbrecht, Germany) was allowed to clot for 30 min in the upright position and then centrifuged for 10 min. The supernatant was then pipetted into three Eppendorf tubes, each with a capacity of 500 µL, and frozen at −80 °C.

Saliva analysis: Saliva analysis was performed at the Institute of Clinical Immunology of the University of Leipzig, Germany. All samples were thawed and subsequently centrifuged at 3000× *g* for 10 min to remove particulate material. Parameters determined in saliva (s-) included the hormones cortisol and testosterone, the cytokines IL-1β, IL-6, IL-8, IL-10, and TNF-α as well as albumin, IgA, α-amylase, aMMP-8, and neopterin. Solid phase enzyme-linked immunosorbent assays (ELISA) by IBL International GmbH, Hamburg, Germany (IgA: REF: DM59171, IL-1β: REF 30149805, IL-6: REF BE58061, IL-8: REF 30149813IL-10: REF BE58101, TNF-α: REF BE58351, aMMP-8: ELH-MMP8-1, neopterin: REF RE59321, α-amylase: REF RE80111, testosterone: REF RE52631, cortisol: REF RE52611) and by Assay Pro (albumin: Cat. No. EA3201-1) were used. The manufacturer’s instructions were followed for the ELISAs, and standard curves were used.

Blood analysis: Blood analyses were performed at Diagnos MVZ GmbH, Berlin and the Institute for Medical Immunology, Charité University Medicine, Berlin, Germany. Parameters determined in the blood (b-) included the hormones testosterone and cortisol, the cytokines IL-1β, IL-6, IL-8, IL-10, and TNF-α, and blood cell counts of erythrocytes, leukocytes, thrombocytes, neutrophils, lymphocytes, monocytes, eosinophils, and basophils, procalcitonin, C-reactive protein (CRP), osmolality, albumin, and α-amylase. Analysis methods included chemiluminescence immunoassays for b-procalcitonin measurement, kinetic-colorimetric test AU-device for b-α-amylase and b-albumin, immunoturbidimetry for b-CRP, and cryometry for b-osmolality, coulter principle for leukocytes, histogram of erythrocytes for erythrocytes and thrombocytes, volume, conductivity, and scatter properties (VCS) for neutrophils, lymphocytes, monocytes, eosinophils, basophils. For cytokine analysis, the MILLIPLEX MAP Human Cytokine/Chemokine Magnetic Bead Pane (Merck Millipore, Darmstadt, Germany) was used.

### 2.4. Statistical Methods

Statistic evaluation was performed using SPSS (Version 24.0, SPSS Inc., Chicago, IL, USA). The metric variables were presented as means and standard deviation (SD). Longitudinal testing between the different time points was performed. For enabling cross-sectional testing of the changes in saliva and blood, ratios of T2 and T3 to baseline values were calculated (T2/T1 and T3/T1) in both saliva and blood. Normal distribution was tested by the Shapiro–Wilk test. The non-parametric Wilcoxon signed-rank test and Friedman test, with Dunn’s pairwise post hoc test in case of significant differences, were used. In the case of concentrations below the detection limit, salivary parameters were corrected for analyzation: s-albumin and s-IL-1β by using half of the given minimal detection limit, s-IL-6 by the minimal standard, and s-α-amylase to 0.1 to avoid division by zero. Pearson correlation of s-/b-cortisol, s-/b-IL-6, s-IgA, s-/b-α amylase, and s-aMMP-8, at all the time points to b-procalcitonin and b-CRP at the corresponding time point was determined. The Pearson correlations of all salivary and blood parameters at all time points to general characteristics and T1-/T2-/T3-b-osmolarity were calculated. All tests were performed two-sided and with a significance level of *p* < 0.05. Multivariate analysis of variance and linear regression were planned for identified significant differences of salivary parameters between the different time points and for identified significant differences of T2/T1 and T3/T1 ratios between saliva and blood to investigate the influence of age, body fat and pace. For cortisol and testosterone, univariate analysis of variance and linear regression were planned for salivary and blood values at T2 depending on the time of day.

## 3. Results

### 3.1. Saliva Analysis

Table 2 presents the salivary parameters at all three time points and the *p*-values for the longitudinal testing. In general, SD was high. There was a significant increase of s-cortisol at T2 (*p*_T1–T2_ = 0.03, *p*_T2–T3_ < 0.01, *p*_T1–T3_ = 0.16). No further longitudinal differences in the absolute saliva values were significant. S-testosterone was decreased at T2 (*p* = 0.26). Looking at cytokines, s-IL-10 and s-TNF-α tests were generally below the detection limit. Non-significantly, s-IL-8 increased after baseline (*p* = 0.10) with the highest mean at T3. The s-IgA was elevated directly after the run (*p* = 0.10) and aMMP-8 increased to T2 and further to T3 (*p* = 0.37).

### 3.2. Blood Analysis

Table 3 presents the blood parameters at the three time points and the *p*-values for longitudinal testing. From T1 to T2 and T2 to T3, various significant differences were determined but not between T1 and T3. B-cortisol was significantly increased (pT1–T2 < 0.01) and b- testosterone significantly decreased at T2 (pT1–T2 < 0.01). For b-IL-1β, all but two samples were below the detection limit, for b-IL-6 four samples at T1 and T3, and for IL-8 one sample. Increased values at T2 were identified for b-IL-6 (pT1–T2 = 0.01), b-IL-8 (pT1–T2 = 0.03) and b-IL-10 (pT1–T2 < 0.01). Regarding the blood cell counts, the number of leukocytes was significantly elevated at T2 (pT1–T2 < 0.01) with a higher proportion of neutrophils (pT1–T2 = 0.02) and lower proportions of lymphocytes (pT1–T2 < 0.01) and eosinophils (pT1–T2 < 0.01). B-procalcitonin (pT1–T2 = 0.03), b-CRP (pT1–T2 < 0.01), and b-osmolality (pT1–T2 = 0.01) were significantly increased at T2.

### 3.3. Salivary and Blood Ratios to Baseline

**Figure 1:**
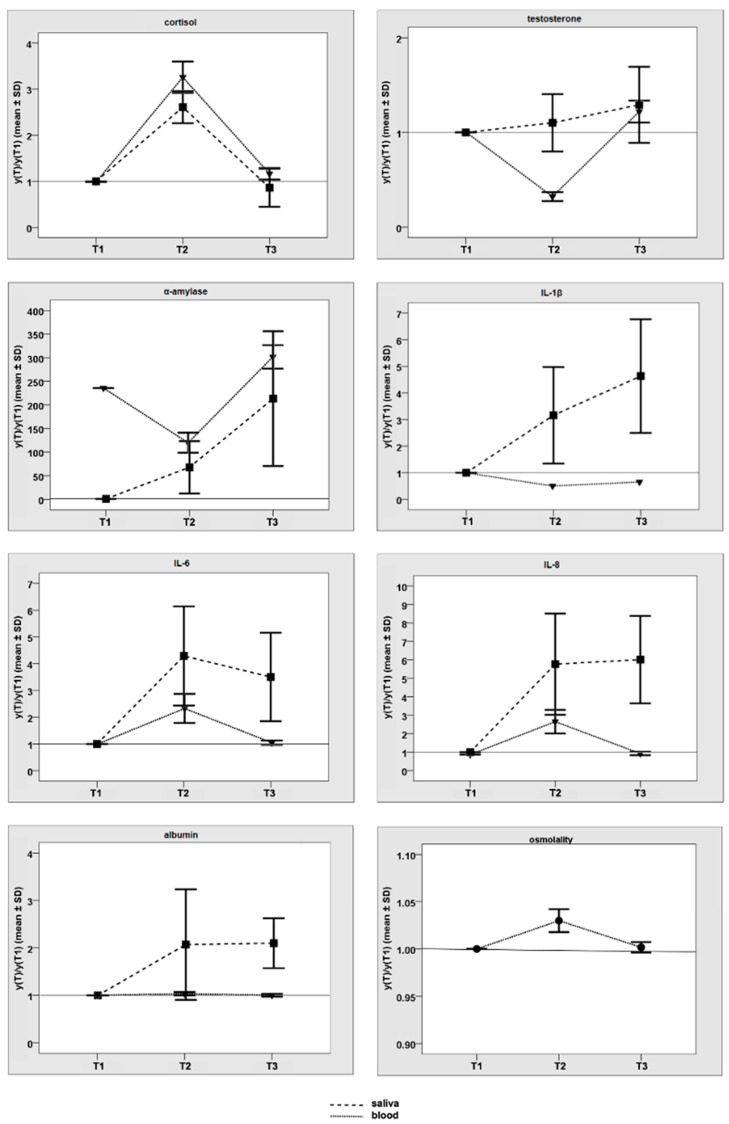
The changes in saliva and blood values from baseline (T1) to shortly after the ultramarathon (T2), and after recovery (T3) are illustrated by the ratios to the baseline values (means and standard deviations of T2/T1 and T3/T1).

In Figure 1, the graphs for the ratios with respect to baseline of both saliva and blood are shown. Exact values and corresponding *p*-values for the comparison of saliva and blood can be retrieved from Table 4. SD was high in general. Significant differences in the longitudinal change between saliva and blood were observed for testosterone (s-T2/T1 = 1.1, b-T2/T1 = 0.3; *p* = 0.02) and α-amylase (s-T2/T1 = 67.8, b-T2/T1 = 0.7; *p* = 0.03).

### 3.4. Associations and Regression Analysis

At baseline, s-cortisol correlated negatively with b-procalcitonin (*p* = 0.019, r = −0.755) and b-CRP (*p* = 0.048, r = −0.67). At T2, b-IL-6 correlated negatively with b-procalcitonin (*p* = 0.037, r = −0.785). No consistent associations between salivary or blood parameters and participants’ characteristics or osmolality were detected.

Multivariate analysis of variance and linear regression was calculated for the difference of T2 to T1 and T2 to T3 of s-cortisol with different models of the parameters age, body fat and pace (Supplementary Table S1). Models with age and body fat, and, respectively, age, body fat and pace, reached statistical significance (T2 − T1: *p* = 0.048; T2 − T3: *p* = 0.003) and linear regression were calculated (T2 − T1: age: β: 0.6, CI95: 0.0–0.1, *p* = 0.057, body fat: β: −0.6, CI95: −0.2–0.0, *p* = 0.057; T2 − T3: age: β: 0.3, CI95: 0.0–0.1, *p* = 0.097, body fat: β: −0.8, CI95: −0.3–−0.1, *p* = 0.002, pace: β: 0.5, CI95: 0.0–0.7, *p* = 0.035). For the differences of T2/T1 ratios between saliva and blood, a significant model for testosterone and the parameters pace and body fat was revealed (*p* = 0.047) but none for α-amylase (*p*_i_ > 0.093, Supplementary Table S1). Neither for b-cortisol, b-testosterone, s-cortisol nor s-testosterone, was a significant influence of the time of day found by ANOVA (*p*_i_ > 0.351, Supplementary Table S1).

## 4. Discussion

Summary of main results: The present pilot study aimed to evaluate changes in blood and salivary parameters in response to an ultramarathon and to ascertain the relation between the results of the two mediums. For various parameters, changes in both blood and saliva could be observed after the ultramarathon. The differences between baseline (T1), shortly after the ultramarathon (T2), and after recovery (T3) were statistically significant for several parameters in blood but only for cortisol in saliva. Comparing the changes between saliva and blood, the ratios to the baseline of testosterone and α-amylase differed significantly.

Discussion in comparison with the literature: In sports medicine, monitoring load and recovery of the athletes is an essential task in daily training [26]. The avoidance of the overtraining syndrome and minimizing injury risk have especially gained recent interest [26,27]. Traditionally, plasma creatine kinase and urea are assessed frequently. However, they do not fulfil the criteria for reliable markers, Creatine kinase especially reflects recent muscle damage [28] and correlates, therefore, with events of collision in contact sports [29]. Urea is highly influenced by recent dietary protein intake [28]. Furthermore, both markers have high interindividual and intraindividual variability [26]. Consequently, identifying further biomarkers for monitoring is of large interest. The International Olympic Committee (IOC) formulated the need for further research to develop and validate measures of load management [27,30].

Salivary assessment is already established as a reliable tool for monitoring stress in biobehavioral research [31,32] and is discussed for diagnosing and monitoring several diseases, such as renal function [33], neurodegenerative diseases [34] and inflammatory bowel diseases [35]. Furthermore, it has now also become of interest in sports medicine because its easy availability allows sampling at many time points to monitor exercise and recovery. A review conducted by Lindsay and Costello presented an overview of studies investigating changes in different salivary parameters as a response to physical exercise [10]. However, often only a few parameters were examined in the same cohort, e.g., immunoglobulins, hormones, or selected interleukins (see below). Furthermore, the relation between salivary parameters and well-described changes in plasma remains rarely investigated [11,12,36,37,38,39,40,41]. The present pilot study provides a comprehensive overview of exercise-induced changes in salivary parameters in comparison to blood changes.

Several studies have described exercise-induced changes for cortisol [1,42,43] and testosterone [42,43,44] as important parts of the hypothalamic–pituitary–adrenal (HPA) axis [10]. The present study showed a significant increase in cortisol levels in both saliva and blood directly after the ultramarathon, whereas only b-testosterone significantly decreased. Such effects of ultraendurance events have also been reported in previous studies [3,8,9,45]. Regarding the changes between pre- and post-exercise, the T2/T1 ratio between saliva and blood did not differ significantly for cortisol but did for testosterone. This result corresponded to a previous study that reported a correlation between the changes from pre- to post-exercise of salivary cortisol and blood cortisol but not of salivary testosterone and blood testosterone [12]. The association between saliva and blood values seems to be less clear for testosterone than for cortisol [11,46]. A significant negative correlation between s-cortisol and b-procalcitonin, as well as b-CRP, at baseline could indicate decreased inflammatory markers because of the immunosuppressive effect of cortisol. Besides the HPA-axis, increased adrenergic activity is part of the psychophysiological response to exercise [47] and leads to increased s-α-amylase concentrations via activation of the salivary glands [48]. This phenomenon could explain the increased salivary T2/T1 ratio for this parameter and the significant difference to its blood values. An increase in s-α-amylase after ultrarunning events has already been described [19,20]. Additionally, also running shorter distances may have such effects on s-cortisol and s-α-amylase with an intensity effect [20,49,50,51]. Despite the revealed significances, the role of confounders must be considered. Multivariate analyses showed a potential influence of age, body fat and pace (Supplementary Table S1).

The s-IgA reflects the mucosal immune status and its reduction after physical exercise was often discussed regarding the risk for upper respiratory tract infections in athletes [2,15,52,53]. Especially, high physical activity was associated with decreased s-IgA and increased incidences of infections [16,17,18]. In contrast, other researchers reported increased s-IgA levels after strenuous exercise [54,55]. In the present study, s-IgA increased not significantly post-exercise. Considering the high SD in the present study and the heterogeneous results of previous studies, a high interindividually different effect and various confounders must be considered. For example, contrary effects for finishers (36 h, decreased s-IgA) and non-finishers of an ultraendurance event (mean 17 h, increased s-IgA) have been reported [56]. Even regarding only ultraendurance events, mainly a decrease has been reported [2,19], while one study showed a non-significant increase [57].

Changes in inflammatory cytokines in response to exercise are well-described for blood [5,6]. Previous studies in (ultra)marathons showed conflicting results: A systematic review states that several studies demonstrated an increase of IL-1β and TNF-α, while other studies reported no changes in the responding levels [5]. Similarly, increases in IL-6, IL-8 and IL-10 were reported [5]. In the present study, no definitive statement concerning b-IL-1β change was possible. In the current study, the other measured cytokines b-IL-6, b-IL-8 and b-IL10, but not b-TNF-α, increased post-exercise, which was in line with available data [5,6]. Regarding the measurement in saliva, the present study could not reveal significant differences. In the literature, no data on ultramarathon runners were available for comparison. In the context of other physical exercises, some studies observed significant effects on salivary interleukins, for example, increases of TNF-α [14] and IL-6 [58], no changes for IL-10 [59], and a significant decrease of IL-1β [60].

Contradictory statements about correlations between saliva and blood values of cytokines are available in the literature [36,37,38,39,40,41]. In the present study, no significant differences between the changes from pre- to post-exercise of IL-1β, IL-6 and IL-8 were identified between saliva and blood. In contrast, one study stated significantly different changes in saliva and blood values in response to high-intensity interval swimming [37]. It is worth mentioning that regarding the changes between two time points, other authors reported significant correlations between the changes in saliva and plasma for IL-1β, IL-6 and TNF-α [61,62]. However, different responses to exercise for IL-6 were reported for serum and salivary IL-6, which could indicate different origins of salivary and systemic IL-6 [40]. In the analysis of salivary cytokines, it can be challenging to achieve proper sampling and detection [63]. Other potential confounders, such as sample collection technique, cold chain management, diurnal variation, and oral health might also play a role [63].

Neopterin can be considered a marker for oxidative stress [64] and its increase in plasma with exercise has been proven in several studies [7]. Additionally, its measurement in urine samples has already been used in sports medicine [1,65]. The present study could also show that an increase in saliva could be measured.

Oral aMMP-8 levels were investigated extensively as a possible diagnostic marker for periodontitis, as this extracellular peptidase takes part in the respective tissue’s degradation [22]. In the present study, s-aMMP-8 was investigated, for the first time, in its dependence on physical exercise and a non-significant increase immediately post-exercise, as well as after recovery, was determined. It remains open to discussion if the increased value affects the periodontal tissue, but increased periodontal inflammation in elite athletes has been reported [66]. Nevertheless, the level remained below the threatening threshold for periodontitis [67] and the relation of salivary aMMP-8 levels to periodontitis is questionable in elite athletes, as a study could not reveal an association in contrast to controls [68].

Strengths and limitations: The wide selection of parameters and analyzing them in both saliva and blood samples were unique to the present study. With participants running more than 160 km, the effect of an intense physical load was evaluated. Such extreme exercise should surely enable detection of exercise-induced changes. As all included participants were able to finish the ultramarathon within 30 h, they could be considered highly trained ultraendurance athletes. This exceptional training level, and the inclusion of only male athletes, ensured a certain comparability, despite big heterogeneity regarding general characteristics. Furthermore, the three time points allowed to assess changes from T1 to T2 and T3, regardless of generally modified inflammatory parameters in this particular population. Nevertheless, several limitations and methodological biases must be addressed. First of all, the small sample size of nine runners must be mentioned. In this small cohort, salivary parameters showed high deviations and only a few parameters met the level of statistical significance. Further studies with more participants are necessary to enable clearer statements. Selection bias could have been caused by the inclusion and exclusion criteria. In this regard, the exclusion of runners that did not finish the ultramarathon should be mentioned. The impact on immunological parameters could have been high because of exhaustion. Their inclusion and comparison to finishers was not possible due to organizational reasons. Further inclusion criteria limited the generalizability of the derived data but enabled a more homogenous cohort with less confounders. Further points of methodological bias must be addressed. Reproducible saliva sampling is a challenge in general and should be performed in accordance with a strict saliva sampling protocol [69]. Moreover, organisational reasons led to inconsistencies in the exact time points of sampling which could be a cause of bias. For example, some parameters, such as cortisol, depend on the time of day [10]. Nevertheless, ANOVA did not show a significant influence of the time of day on both salivary and blood cortisol and testosterone. It can be difficult, especially directly after such intense exercise, to obtain immediate sampling, as well as to avoid confounders, such as drinking. For each participant, sampling at T2 took place within 30 min after finishing. Other possible confounders were oral health [70,71], saliva collection method (e.g., unstimulated versus stimulated saliva [63], cotton-interference effects [41]), psychological conditions [21,72] and BMI [73], which could have influenced the results and/or impaired the comparability to other data. For interpretation in relation to other data, it must be considered that some studies adjusted the measured values by total protein, osmolality, or saliva flow rate to reduce the effect of exercise-induced dehydration [10,74]. Regarding the results of the present study, several measurements were below the detection limit, in particular cytokine levels. Consequently, s-IL-10, and s-TNF-α cannot be interpreted in the present study and s-albumin, s-α-amylase, s-IL-6, and s-IL-1β must be interpreted with caution as values below the detection limit were corrected for analysis. The reasons for these low values remain unclear. A possible explanation could be storage conditions between saliva collection and analysis [38,63], despite adequate freezing in the present study. It is worth mentioning that other studies also reported that they did not detect IL-10 in saliva [75]. For future studies, strict saliva sampling protocols, sensitive assays, and immediate analysis should be considered for these parameters.

## 5. Conclusions

The present pilot study measured a wide selection of salivary parameters and provides a direct comparison to blood parameters. Various blood parameters and salivary cortisol showed statistically significant changes in response to an ultramarathon. The blood parameters, cortisol, testosterone, IL-8, IL-6, and α-amylase offer potential for innovative monitoring of exercise and recovery. Most salivary parameters changed in analogy to blood markers, but some (testosterone and α-amylase) differed significantly. Consequently, for proper interpretation of salivary parameters, saliva-specific reference values need to be determined and the role of confounders must be addressed in future studies.

## Figures and Tables

**Table 1:** Characteristics of the participants.

*n* = 9	Mean ± SD	Range
age (years)	48 ± 8.8	35–62
height (m)	1.76 ± 0.07	1.69–1.86
weight (kg)	72.5 ± 7.2	61.2–85.2
body fat (%)	16.65 ± 4.93	10.7–23.87
BMI (kg/cm²)	23.53 ± 1.90	21.3–27.65
running time (h)	23:29 ± 03:58	-
pace (min/km)	08:42 ± 01:28	-

Abbreviations: BMI, body mass index; *n*, number of participants; SD, standard deviation; -, range not stated to maintain anonymity of the participants.

**Table 2:** Salivary parameters at baseline (T1), shortly after the ultramarathon (T2), and after recovery (T3) and *p*-value for differences between time points. The *p*-values indicate the longitudinal differences calculated by the Friedman test and, in case of significant differences, by Dunn’s pairwise post hoc test. Bold values and an asterisk mark the significant differences (*p* < 0.05).

Parameter	T1 (Mean ± SD)		T2 (Mean ± SD)		T3 (Mean ± SD)		T1 vs. T2	T2 vs. T3	T1 vs. T3
s-cortisol (µg/dL)	0.7	±0.2	1.8	±0.7	0.6	±0.7	0.03 *	<0.01 *	0.16
s-testosterone (pg/mL)	325.3	±248.6	298.3	±262.0	313.4	±291.0	0.26	0.26	0.26
s-IL-1β (pg/mL)	6.2	±8.7	6.6	±10.4	9.4	±12.2	0.87	0.87	0.87
s-IL-6 (pg/mL)	0.7	±1.1	0.9	±0.7	0.8	±1.3	0.46	0.46	0.46
s-IL-8 (pg/mL)	28.6	±39.7	59.9	±60.5	145.8	±218.0	0.10	0.10	0.10
s-IL-10 (pg/mL)	<OOR	<OOR	<OOR	<OOR	<OOR	<OOR	n.c.	n.c.	n.c.
s-TNFα (pg/mL)	<OOR	<OOR	<OOR	<OOR	<OOR	<OOR	n.c.	n.c.	n.c.
s-IgA (µg/mL)	11.6	±11.4	32.4	±61.4	16.4	±7.7	0.10	0.10	0.10
s-α amylase (U/mL)	18.4	±20.6	31.2	±26.5	33.3	±39.9	0.46	0.46	0.46
s-aMMP-8 (pg/mL)	1749	±1614	7422	±8900	14834	±23062	0.37	0.37	0.37
s-neopterin (nmol/L)	2.6	±1.4	3.7	±2.1	2.8	±1.5	0.37	0.37	0.37
s-albumin (ng/mL)	9.4	±7.0	8.5	±6.7	17.7	±19.5	0.24	0.24	0.24

Abbreviations: aMMP-8, active-matrix metalloproteinase-8; IL, interleukin; n.c., not calculable; <OOR, out of range: below detection limit; s, salivary; SD, standard deviation.

**Table 3:** Blood parameters at baseline (T1), shortly after the ultramarathon (T2), and after recovery (T3) and *p*-values for differences between time points. The *p*-values indicate the longitudinal differences calculated by Friedman test and, in case of significant differences, by Dunn’s pairwise post hoc test. Bold values and an asterisk mark the significant differences (*p* < 0.05).

Parameter	T1 (Mean ± SD)		T2 (Mean ± SD)		T3 (Mean ± SD)		T1 vs. T2	T2 vs. T3	*p* T1 vs. T3
b-cortisol (µg/dL)	9.7	±2.5	30.7	±8.1	10.8	±2.1	<0.01 *	<0.01 *	0.81
b-testosterone (pg/mL)	3652	±915	1129	±467	4283	±1106	<0.01 *	<0.01 *	0.48
b-IL -1β (pg/mL)	8.9	±7.7	5.5	±2.5	9.4	±0.0.	n.c.	n.c.	n.c.
b-IL-6 (pg/mL)	7.3	±4.8	13.5	±8.5	6.7	±3.7	0.01 *	0.08	0.48
b-IL-8 (pg/mL)	10.8	±9.6	26.6	±17.5	11.8	±11.6	0.03 *	0.02 *	0.89
b-IL-10 (pg/mL)	9.8	±6.0	28.0	±15.4	10.2	±6.6	0.01 *	0.14	0.29
b-TNFα (pg/mL)	7.4	±1.7	7.6	±1.7	6.6	±1.8	0.44	0.44	0.44
b-α amylase (U/mL)	0.07	±0.02	0.05	±0.01	0.08	±0.02	0.01 *	<0.01 *	0.24
b-erythrocytes (M/µL)	4.45	±0.34	4.58	±0.42	4.48	±0.38	0.90	0.90	0.90
b-thrombocytes (k/µL)	226	±34	222	±47	248	±54	0.12	0.12	0.12
b-leucocytes (g/dL)	5.2	±1.4	10.9	±3.0	5.4	±1.0	<0.01 *	<0.01 *	0.64
b-neutrophils (%)	59.1	±8.0	75.6	±13.6	56.8	±10.5	0.02 *	<0.01 *	0.35
b-lymphocytes (%)	30.1	±8.2	13.1	±8.4	30.9	±11.1	<0.01 *	<0.01 *	0.48
b-monocytes (%)	8.0	±1.4	10.4	±5.9	9.3	±1.4	0.26	0.26	0.26
b-eosinophils (%)	2.1	±1.6	0.2	±0.4	2.3	±1.4	<0.01 *	<0.01 *	0.81
b-basophils (%)	0.8	±0.2	0.7	±0.3	0.7	±0.5	0.90	0.90	0.90
b-procalcitonin (µg/L)	0.1	±0.1	0.6	±0.3	0.2	±0.4	0.03 *	<0.01 *	0.64
b-C-reactive protein (mg/L)	3.1	±4.4	34.7	±18.7	4.2	±5.7	<0.01 *	<0.01 *	0.81
b-albumin (ng/mL)	43.8	±2.8	45.2	±4.2	43.7	±2.1	0.46	0.46	0.46
b-osmolality (mOsmol/kg)	290.0	±4.6	298.1	±7.8	290.0	±2.3	0.01 *	0.02 *	0.81

Abbreviations: IL, interleukin; n.c., not calculable; s, salivary; SD, standard deviation; TNF-α, tumor necrosis factor alpha.

**Table 4:** Longitudinal change expressed as ratios of T2 and T3 to baseline of salivary and blood parameters. The *p*-values indicate the differences between saliva and blood calculated by Wilcoxon signed-rank test. Bold values and an asterisk mark the significant differences (*p* < 0.05).

Parameter	T2/T1 Ratio					T3/T1 Ratio				
	Saliva		Blood		*p*-Value	Saliva		Blood		*p*-Value
	(Mean ± SD)		(Mean ± SD)			(Mean ± SD)		(Mean ± SD)		
cortisol	2.6	±1.0	3.3	±1.0	0.09	0.9	±1.2	1.2	±0.4	0.11
testosterone	1.1	±0.9	0.3	±0.1	0.02 *	1.3	±1.2	1.2	±0.3	0.86
IL-1β	3.2	±5.4	0.8	±0.4	0.66	4.6	±6.4	0.7	±0.0	n.c.
IL-6	4.3	±5.6	2.3	±1.1	0.07	3.5	±5.0	1.0	±0.2	0.23
IL-8	5.8	±8.2	2.7	±1.6	0.13	6.0	±7.1	1.1	±0.3	0.05
IL-10	n.c.	n.c.	3.1	±2.3	n.c.	n.c.	n.c.	1.0	±0.16	n.c.
TNFα	n.c.	n.c.	1.1	±0.3	n.c.	n.c.	n.c.	0.9	±0.24	n.c.
α-amylase	67.8	±166.2	0.7	±0.2	0.03 *	213.4	±428.4	1.2	±0.2	0.86
albumin	2.1	±3.5	1.0	±0.1	0.86	2.1	±1.6	1.0	±0.1	0.07

Abbreviations: IL, interleukin; n.c., not calculable; s, salivary; SD, standard deviation.

## Data Availability

The data supporting this study’s findings are available from the corresponding author upon reasonable request.

## Supplementary Materials

The following supporting information can be downloaded at: https://www.mdpi.com/article/10.3390/ijerph192316110/s1, Table S1. Results of multivariate analysis of variance and linear regression for the difference between T2 and T1 and T2 and T3 of salivary cortisol (s-cortisol T2-T1), for the difference of T2/T1 ratios between salivary and blood testosterone (T2/T1 testosterone s-b) and α-amylase (T2/T1 α-amylase s-b) with the predictors age, body fat and pace; and of univariate analysis and linear regression for salivary and blood cortisol (T2 s-cortisol, T2 b-cortisol) and testosterone at T2 (T2 s-testosterone, T2 b-testosterone) with the predictor time of day.
