# Supplementary material for: Salivary Diagnostic for Monitoring Strenuous Exercise—A Pilot Study in a Cohort of Male Ultramarathon Runners
Source: Int J Environ Res Public Health. 2022 Dec 1;19(23):16110. doi: 10.3390/ijerph192316110 (PMC9737684; doi:10.3390/ijerph192316110)
Supplement: Supplementary file 1 [file ijerph-19-16110-s001.zip › ijerph-2010913-supplementary.pdf]

Salivary diagnostic for monitoring strenuous exercise – a pilot study in a cohort of male ultramarathon runners

Table S1. Results of multivariate analysis of variance and linear regression for the difference between T2 and T1 and T2 and T3 of salivary cortisol (s-cortisol T2-T1), for the difference of T2/T1 ratios between salivary and blood testosterone (T2/T1 testosterone s-b) and α-amylase (T2/T1 α-amylase s-b) with the predictors age, body fat and pace; and of univariate analysis and linear regression for salivary and blood cortisol (T2 s-cortisol, T2 b-cortisol) and testosterone at T2 (T2 s-testosterone, T2 b-testosterone) with the predictor time of day.

Dependent variable	Model	Predictors	β	Confidence Interval (95 %)		p-Value
				Lower Bound	Upper Bound	
s-cortisol T2-T1	1					0.132
		pace (min/km)	0.7	0.0	0.1	0.135
		body fat (%)	-0.6	-0.2	0.0	0.121
		age (years)	-0.2	-0.6	0.4	0.665
	2					**0.048***
		age (years)	0.6	0.0	0.1	0.057
		body fat (%)	-0.6	-0.2	0.0	0.057
s-cortisol T2-T3	1					**0.003***
		age (years)	0.3	0.0	0.1	0.097
		body fat (%)	-0.8	-0.3	-0.1	**0.002***
		pace (min/km)	0.5	0.0	0.7	**0.035***
T2/T1 testosterone s-b	1					0.116
		age (years)	0.3	-0.1	0.1	0.506
		body fat (%)	0.6	0.0	0.3	0.084
		pace (min/km)	-1.0	-1.2	0.1	0.065
	2					**0.047***
		body fat (%)	0.6	-0.0	0.2	0.071
		pace (min/km)	-0.8	-0.8	-0.1	**0.027***
T2/T1 α-amylase s-b	1					0.223
		age (years)	-0.3	-29.2	18.3	0.562
		body fat (%)	0.4	-17.6	46.5	0.279
		pace (min/km)	0.7	-62.6	224.3	0.193
	2					0.106
		body fat (%)	0.5	-10.8	43.3	0.184
		pace (min/km)	0.5	-27.2	140.8	0.143
	3					0.093
		pace (min/km)	0.6	-15.9	155.4	0.093
T2 s-cortisol	1	time of day by running time (h)	0.3	-0.1	0.2	0.430
T2 b-cortisol	1	time of day by running time (h)	0.4	-2.4	1.0	0.352
T2 s-testosterone	1	time of day by running time (h)	-0.3	-74.3	39.5	0.493
T2 b-testosterone	1	time of day by running time (h)	-0.1	-119.3	89.1	0.742
